# Evaluation with structural equation modeling of variables affecting health-seeking behaviors during the COVID-19 pandemic in İstanbul, Türkiye

**DOI:** 10.55730/1300-0144.5878

**Published:** 2024-07-12

**Authors:** Pınar ATALI, Seyhan HIDIROĞLU, Alican SARISALTIK, Melda KARAVUŞ

**Affiliations:** 1 Tuzla District Health Directorate, Republic of Türkiye Ministry of Health, İstanbul, Turkiye; 2 Department of Public Health, Faculty of Medicine, Marmara University, İstanbul, Turkiye; 3 Çayırova District Health Directorate, Republic of Türkiye Ministry of Health, Kocaeli, Turkiye

**Keywords:** COVID-19, pandemic, health-seeking behavior, structural equation modeling

## Abstract

**Background/aim:**

The relation between the demographic characteristics of individuals and their health-seeking behaviors was presented and the effects of health cognitions, healthy lifestyle behaviors, and coronavirus fear levels on health-seeking behaviors during the COVID-19 pandemic were examined.

**Materials and methods:**

This descriptive survey study was conducted in the Tuzla District of İstanbul, Türkiye, between March and June 2021.

**Results:**

From analysis of the 391 participants, 60.0% were females, 27.1% were between 31 and 40 years of age, 47.0% were healthcare professionals, and the perceived socioeconomic status of 50.9% was above average. According to the results, the women exhibited more health-seeking behavior than the men (p < 0.05). While the young participants showed more online health-seeking behavior (p < 0.05), the older ones showed greater health responsibility (p < 0.05). The participants with a high level of education exhibited traditional health-seeking behavior (p < 0.05) more than the others, and below-low socioeconomic status increased the COVID-19 fear level 1.94 times (95.0% CI: 1.08–3.48). The Health-Seeking Behavior Scale (HSBS) score was related to the Health Cognitions Questionnaire (HCQ) (p < 0.0001) and the Healthy Lifestyle Behaviors Scale-II (HLBS-II) scores (p = 0.002; Table 3). While the HSBS score was positively associated with an increase in the HCQ score and HLBS-II score (p < 0.05), the HSBS score was not significantly related to the Fear of COVID-19 Scale score (p > 0.05).

**Conclusion:**

While fear of COVID-19 was not significantly influential, health cognitions and healthy lifestyle behaviors were the main factors that led to health-seeking behavior during the COVID-19 pandemic.

## 1. Introduction

Health-seeking behavior can be defined as actions taken to solve current and potential health problems by professional means. Humanity throughout its existence has been experiencing constant change caused by events in its natural environment. One natural event that humanity has encountered many times to date is pandemics. Throughout history, pandemics have affected states, societies, and individuals. It is known that there is a relation between how individuals perceive a disease and their response and adaptation to it [1]. For this reason, the health-seeking behavior of individuals in response to a threat is important to minimize the speed of spread of an epidemic to reduce its geographical prevalence and possible loss of life. In addition, knowledge of individuals’ response to a threat is essential for authorities to be able to handle epidemics and plan accordingly [2].

It should be regarded as the responsibility of public health professionals to understand how society relates to measures because of the current extraordinary conditions and changing daily life during the coronavirus disease 2019 (COVID-19). For this purpose, recent studies have shown important results concerning the variables that shape health behaviors regarding compliance with the measures taken. One of these studies, How Democracies Cope with COVID-19: A Data Driven Approach, 2020–2021 (The HOPE Project), was a scale study published by a group of Danish academics. The aim of their study was to reveal and compare how citizens in Western countries approach the measures taken regarding the pandemic. The study had a mission to transparently explain how the COVID-19 restrictions were implemented in Denmark to maintain citizens’ trust in their government. Thus, the Danish Health Authority had an opportunity to collect all individuals’ posts, comments, and responses to comments, and the data collected were used for scientific purposes only. It was hoped to be seen as a general social example of communication of information during a health crisis. The science of health communication has highlighted the need to advance science informatics over the last decade by building trust through transparency [3]. The HOPE Project contributed to Denmark’s relatively successful management of the pandemic’s early stages; the first results of the survey conducted among 26,508 people showed that threat perception and fear were the determining factors, even across cultures, in line with the measures taken [4]. Another significant result from the HOPE Project reveals that believing in the effectiveness of measures was a strong factor that positively affects health-seeking behaviors [4]. Moreover, it has been observed that the effects of this belief were powerful on individuals with weak threat perception and this suggests that the health belief model that was not based on health anxiety or fear [4].

Research on this subject in Türkiye was carried out relatively early in the pandemic. Long-term, repeated field studies are needed to reveal the factors that shape the health behavior of the public. The question concerning the ‘need for field studies produced in the field of health-seeking behavior’ can be answered within the framework of ‘effectiveness’.

### 1.1. Study content

In our research, in addition to examining the relation between individuals’ demographic data and health-seeking behavior, structural equation modeling (SEM) was performed to examine the effect level of health cognitions, fear of COVID-19, and healthy lifestyle behaviors on health-seeking behavior. If individuals who do not have health concerns do not show health-seeking behavior, this situation can cause serious health problems in the future. On the other hand, if individuals with high health concerns use health services unnecessarily, people really needing them can be prevented from accessing them. When individuals have high levels of health-seeking behaviors and healthy lifestyle behaviors, they do not worry excessively and can maintain the balance. One obvious thing was that the COVID-19 pandemic caused a widespread effect on the mental well-being of people across the globe [5].

### 1.2. Multivariate model analysis

Multivariate model analyses show the level of effect of a change in each independent variable on the outcome and determines the level of its effect. SEM is a second-generation method of data analysis, addressing a research question by modeling the connections among multiple independent and dependent variables [6]. A recent SEM study that examined the relation between fear and anxiety related to COVID-19 among pregnant women showed that COVID-19-induced anxiety had indirect effects on the mental well-being of pregnant women [7]. It has been seen that modeling studies facilitate the complex analysis created with dynamic latent variables in health research. In the literature, similar modeling studies in the field of health conducted abroad mostly examine the effects of health behaviors, quality of life, anxiety, and risk factors in individuals with chronic diseases. For example, a study was conducted to evaluate the impact of the Mediterranean diet in moderating the negative impact of depression and anxiety on the likelihood of developing cardiovascular disease with a structural equation modeling approach [8]. Generalized, behavioral examples are the basis of modeling studies, especially in health. However, as modeling studies in Türkiye have been limited, our research aimed to provide access to more useful scientific data to understand the variables’ effects and their effectiveness levels on health-seeking behaviors for health policies carried out in this field.

## 2. Materials and methods

This descriptive survey study was conducted in the Tuzla District of İstanbul, Türkiye. The research population, which consisted of adults over aged 18 living in Tuzla, numbered 85,446.

### 2.1. Study design

Although our study involved descriptive research, the relation of individuals’ demographic characteristics with health-seeking behavior and the effect of health cognition, healthy lifestyle behaviors, and coronavirus fear level on health-seeking behavior were in a cross-sectional analytical design. Since those who volunteered to participate in the study were included in the sample using the convenient sampling method (not a probability sampling method), we could not generalize the results to the entire adult population of Tuzla, which hindered the cross-sectional analytical design of our study. Since confirmatory factor analysis and correlations between scales were investigated, our study also had a methodological design section.

### 2.2. Sample size

During the data collection period of March–June 2021 the individuals contacted for filiation studies of COVID-19 and vaccination studies in the research area were invited to participate in our survey. A total of 391 people volunteered. Since there were missing questions (unanswered), the sample number (n) was not given as 391 on all scales.

### 2.3. Data collection

The data were collected via questionnaires, printed and online, in a way that all participants could understand much more easily by taking care to answer in a period appropriate to the research topic. Previously validated scales were utilized and their comprehension and acceptability were ensured among all the participants. Due to the pandemic, an online survey was sent to those who wanted to participate in the research from among those who visited during the filiation studies. The questionnaire was applied to the elderly group during the vaccination studies at home. Printed questionnaires were given at health institutions and public places for those who wanted to participate in the study. The questionnaire consisted of five parts, with a total of 108 questions. The initial section of the data collection form included items regarding the demographic characteristics of the participants. The Health Seeking Behavior Scale (HSBS), the Health Cognitions Questionnaire (HCQ), the Healthy Lifestyle Behaviors Scale-II (HLBS-II), and the Fear of COVID-19 Scale (FCV-19S) were applied in the second part of the data collection form.

#### 2.3.1. Health Seeking Behavior Scale

This scale, developed by Kıraç and Öztürk in 2021, consists of 12 items and three subdimensions [9]. The online health-seeking behavior subdimension contains six items, while the traditional and professional subdimensions each have three items. Cronbach’s alpha coefficient was 0.755 for the HSBS. The items are rated on a 5-point Likert-type scale ranging from “strongly disagree = 1” to “strongly agree = 5.” The total scores from the scale vary between 12 and 60. High scores reflect a high level of health-seeking behaviors [9].

#### 2.3.2. Health Cognitions Questionnaire

This questionnaire, developed by Hadjistavropoulos et al. in 2012, evaluates dysfunctional health-related beliefs associated with the severity of health anxiety experienced by individuals [10]. The scale consists of 20 items prepared by the Likert method ranging from “strongly disagree = 1” to “strongly agree = 5.” The total score obtained from the scale varies between 20 and 100. The scale consists of four subdimensions: possibility of disease, severity of the disease, difficulty in coping with the disease, and inadequacy of medical services. High scores on the scale reflect high dysfunctional beliefs about health [11, 12]. Cronbach’s alpha coefficient was calculated as 0.821 [12].

#### 2.3.3. Healthy Lifestyle Behaviors Scale-II

The HLBS was developed in 1987 and revised into the HLBS-II by Walker in 1996 [13]. The Turkish version of the scale was developed by Bahar et al. in 2008 [14]. The HLBS-II is a 4-point Likert-type scale (“never = 1” to “regularly = 4”) and consists of 52 items and six subdimensions. The subdimensions include physical activity, nutrition, spiritual growth, interpersonal support, health responsibility, and stress management. Cronbach’s alpha coefficient of the HLBS-II was 0.92 [14]. The total scores vary from 52 to 208. Higher scores show a more common practice of healthy behaviors [14].

#### 2.3.4. The Fear of COVID-19 Scale

This scale, developed by Ahorsu et al., consists of seven items and is unidimensional [5]. The validity and reliability of the Turkish version of the FCV-19S were confirmed by Bakioğlu et al. [15]. Cronbach’s alpha coefficient of the FCV-19S was 0.82. The items were answered on a 5-point Likert scale (“strongly disagree = 1” to “strongly agree = 5”). The scores from the scale vary between 7 and 35. Higher scores show a high level of fear towards the coronavirus [15].

### 2.4. Statistical analysis

SPSS for Windows version 21.0 (IBM Corporation, Armonk, NY, USA, released 2012) was used to analyze the data obtained for our study. The HCQ, HLBS-II, and FCV-19S scores’ predicted effects on the HSBS score as the dependent variable were examined by SEM with the STATA 17 statistical software for data science.

Although our study was a descriptive study with a cross-sectional analytical design, we wanted to test our data-collecting tools by adding a methodological design. The HCQ, HSBS, FCV-19S, and HLBS-II scores were the independent variables of the study; in the structural equation model only health-seeking behavior was the dependent variable. The independent variables of the study were demographic characteristics: age group, sex, educational level, occupation, marital status, employment status, the region where they lived longest in the last five years, family type, the region where they lived until the age of 12, perceived socioeconomic status, educational level of parents, presence of ever positive COVID-19 polymerase chain reaction (PCR) test result, chronic disease, medication use, health insurance, and the status of the first health institution presented to when necessary. Descriptive statistics were presented as percentages and median and range (minimum–maximum) values. Continuous variables were tested by histogram graphics and the Kolmogorov–Smirnov test for normal distribution. The Mann–Whitney U and Kruskal–Wallis tests were used to compare the differences of the scale and the subscales scores in the individual characteristics. In addition, independent variables and scale scores relations were evaluated with logistic regression analysis. Scale scores were dichotomized according to the median values of the study group and considered the dependent variable in the logistic regression analyses.

Furthermore, categories of the region of the residence until the age of 12, educational level, and socioeconomic level variables were reduced for further evaluation in the logistic regression analysis. Villages and towns were considered small settlements, city and district centers were considered large settlement centers, and others were considered abroad. The educational level variable was categorized as lower than high school (illiterate, literate, primary school, and secondary school), high school, and higher education (high school, associate degree, and graduate degree). Socioeconomic status was reduced into three categories for logistic regression analysis in which below low-level and above-low level were categorized as low socioeconomic status, while below average and above average were categorized as middle socioeconomic status. High- and top-level socioeconomic status were categorized as high socioeconomic status.

### 2.5. Ethical issues

Permission to conduct the research was obtained from the Ministry of Health of the Republic of Türkiye COVID-19 Platform of the Directorate General. The study was confirmed to be in line with the Declaration of Helsinki by the Clinical Trials Ethics Committee of Marmara University School of Medicine (06.11.2020/09.2020.1212).

## 3. Results

A total of 236 (60.4%) of the participants were females and most of the participants were aged 31–40 years (n = 106, 27.1%) or 41–50 years (n = 105, 24.3%). In total 62 (15.9%) of the participants had at least one positive COVID-19 PCR test. A total of 243 (62.3%) had received higher education and 274 (70.1%) were working. Most of the occupational groups in the study were healthcare professionals (n = 136, 47.0%) and educators (n = 49, 17.1%). The participants were mainly from nuclear families (n = 316, 80.8%). Half of the participants’ perceived socioeconomic status was above average (n = 199, 50.9%). Approximately one-fourth of the participants had a chronic disease (n = 101, 25.8%) and took medications continuously (n = 109, 28%). Half of the participants used the family medicine unit as their first choice for healthcare (n = 204, 52.3%) and 28.5% (n = 111) the state hospital. The median scores of the participants obtained from each scale are presented in Table 1. Since there were missing data on some scale items, n was not given as 391 in all scales.

The median online health-seeking behavior subdimension scores of the HSBS were 19.0 (6–30) higher in females and in the participants aged 26–30 (22.0, range 11–30). The median traditional health-seeking behavior score was 10 (3–15) higher in the participants who had graduated from high school and had received higher education. The median professional health-seeking behavior score was 12 (5–15) higher in the participants who had a secondary school education and in those who had an elementary school education (13, range 5–15). Growing up in a town until the age of 12 (OR: 2.46, 95.0% CI [1.01–5.99], p < 0.05) and in the district center (OR: 1.87, 95.0% CI [1.10–3.50], p < 0.05) was associated with a higher HSBS total score. Mothers participating in the study who had an elementary school education (OR: 2.20, 95.0% CI [1.22–3.97], p < 0.05) and fathers participating who had a secondary school education (OR: 4.14, 95.0% CI [1.33–12.8], p < 0.05) had higher HSBS total scores. It was found that the other individual characteristics did not affect the HSBS score significantly in the logistic regression analysis (Table 2).

The subdimension of severity of the disease was higher (13, range 4–20) in females and the participants aged 21–25 (15, range 10–20). The median difficulty in coping with the disease subdimension was higher in the participants over the age of 70 (24, range 16–32). Moreover, the participants with below low-level socioeconomic status had a higher level of difficulty in coping with the disease (25, range 16–33). According to the results, living in a town and in the district center until the age of 12 was associated with a higher HCQ total score (OR: 3.08, 95.0% CI [1.25–7.57], p < 0.05), (OR: 2.66, 95.0% [CI = 1.41–5.03], p < 0.05). The other independent variables were not statistically significant in the logistic regression analysis according to the HCQ score (Table 2).

The participants whose first preference of health institution to present to was the family medicine unit (139, range 82–205) and the state hospital (135.5, range 82–199) showed significantly higher scores than the participants who presented to any other health institution (132.5, range 70–204) on the total score of the HLBS-II. The median health responsibility subdimension score was 24 (13–36) higher in females. Moreover, the nutrition (22, range 12–35) and the interpersonal support (27, range 13–36) subdimension scores were higher in the females than in the males. The median health responsibility subdimension score was 27 (16–34) higher in the participants aged 61–70 years. The health responsibility of the participants who lived in a village until the age of 12 (25, range 14–36) and those who lived in a town until the age of 12 (25.5, range 14–33) was significantly higher than that in the other participants. In the age groups of 31–40 (OR: 2.21, 95.0% CI [1.10–4.42], p < 0.05) and 61–70 (OR: 6.75, 95.0% CI [1.31–35.01], p < 0.05) higher HLBS-II total scores were found. In addition, having a perceived socioeconomic status above average increased the HLBS-II total score (OR: 6.00, 95.0% CI [1.29–28.40], p < 0.05). The other individual characteristics were not significant in the logistic regression analysis of the HLBS-II score (Table 2).

The FCV-19S scores were higher in the participants who were female (17, range 7–35), working in nonhealth professions (17, range 7–35), and had below low-level socioeconomic status (18.5, range 9–27). Being female (OR: 1.45, 95.0% CI [1.12–1.89], p < 0.05), not working in the healthcare field (OR: 1.78, 95.0% CI [1.11–2.87], p < 0.05), and having below-low socioeconomic status (OR: 1.94, 95.0% CI [1.08–3.48], p < 0.05) were associated with higher FCV-19S total scores. The other individual characteristics were not significant in the logistic regression analysis of the FCV-19S score (Table 2).

The standardized root mean squared residual (SRMR) and coefficient of determination (CD) compliance criteria of all scales showed acceptable values (Table 4). The data we provided determined that the model used for our research fit well according to SRMR and CD criteria (Figure). On structural equation modeling, HSBS score was found to be related to HCQ (p < 0.0001) and HLBS-II scores (p = 0.002, Table 3). That is, an increase in HSBS score was positively associated with an increase in HCQ and HLBS-II scores, whereas HSBS score was not significantly related to FCV-19S score (p > 0.05).

## 4. Discussion

The high participation of females in our study can be explained by the fact that they engage in more health-seeking behavior than males. A previous study on personal health responsibility determined that females acted more responsibly towards their health [16]. The results of our study indicated that females’ online health-seeking behavior, disease severity, health responsibility, nutrition, and interpersonal support levels were higher than those of males. Generally, women have longer life expectancy than men, get sick more frequently, and benefit from health services at a higher level [17]. According to the logistic regression analysis of our study, the COVID-19 fear level of adult women was 1.45 times higher than that of men. Research has revealed that women tend to experience more fear towards COVID-19 compared to men, potentially due to gender-based variations in sensitivity and susceptibility to stress. Furthermore, women may face an increased likelihood of developing mental health issues following stressful life events [18].

According to our study, while online health-seeking behavior was more prevalent in those aged 26–30, those aged 61–70 had a higher tendency to take responsibility for their health. This can be explained by the fact that online computer and/or mobile device use can be more accessible for the young participants. In contrast, with increasing age, individuals might become more sensitive about their health and fulfill their health responsibilities better [18]. A study that examined the impact of COVID-19 threat perception and new media literacy on e-health literacy using path analysis found e-health to be a large part of health literacy [19]. As most of the participants were young people (37% were 25 and below and 47% were 26–40), as in our study, they mainly obtain health-related information through digital media. Another finding obtained in their research was that COVID-19 threat perception did not significantly affect e-health literacy [19]. Our study found no statistically significant relation between having a positive COVID-19 PCR test result and health cognition, health-seeking behavior, fear of COVID-19, or healthy lifestyle behaviors. A similar study indicated no significant difference in any aspect of health-seeking behaviors, health perception, certainty, the importance of health, or self-awareness levels with suffering COVID-19 disease or not [20]. Other model research showed that eliminating uncertainty from the fear of COVID-19 will contribute to reducing depression, anxiety, and stress and increasing positivity [15].

However, according to another previous study, the most influential factors in determining positive health perception were marriage, education, and income [21]. In the structural equation model on factors affecting health-seeking behaviors, a significant relation was detected between the participants’ educational level and their health literacy and online health-seeking behavior. In our research, the online health-seeking behavior level of the illiterate and primary school educated participants was lower than that of those with a high school, associate, graduate, and higher graduate education. In a previous study, the professional health-seeking behavior of participants with a high school education was lower than that of those with higher education [4]. Our study revealed that participants with a high school and higher education were more likely to engage in traditional methods of seeking healthcare. However, in our study the participants who did not have a high school education or higher were more likely to engage professional health-seeking behavior. Individuals who have limited education do not have any alternative to benefit from other than government health services and might not be able to find a source to ask or trust other than the professional health provider; therefore, they could consult professionals more often.

The concept of family, which is a social unit, shapes the members of that society with its structure, function, and needs. The World Health Organization (WHO) puts the family at the center of improving the health of individuals. In our study we did not observe a relation between family structure and health-seeking behaviors. Based on the findings of a comparable study, there was no notable distinction between family composition and the use of online, professional, or traditional methods for seeking healthcare [22]. According to the cognitive-behavioral approach, maladaptive basic beliefs developed in childhood are thought to be based on health anxiety [23]. These maladaptive beliefs are thought to arise from the individual’s past experiences, the teachings that he or she has experienced in the close family, or the disease processes of the sick parent [24].

Growing up in urban spaces brings with it many difficulties [25]. In a study conducted to determine whether some variables like sex, number of siblings, age of the mother, relationship with siblings, participation in social activities, and the situation reading books in the social skills of children living in the village, a significant difference was found [25]. Another study revealed that age, grade level, birth order, father’s age, parental education level, family type, perceived parental attitude, active participation in classes, and receiving support when faced with a problem did not make a significant difference [26]. The study also showed no significant difference in any of the variables listed in the social skills of children living in the city [26]. The findings from our study indicate that individuals who spent their formative years in urban environments had more dysfunctional health beliefs, whereas those raised in villages and towns demonstrated a stronger sense of health responsibility. More significant results can be achieved when the effects of rural and urban life on health anxiety are examined together with the other components of the sociocultural structure, such as family structure, parental education level, and socioeconomic level. Studies in Türkiye showed that parents’ attitudes towards children differ according to their socioeconomic and educational levels. The data showed that an increase in parental education led to a rise in democratic attitudes towards their children, while overprotective and strict disciplinary attitudes decreased [27]. Another accepted point is that poverty was the most determining risk factor for health [28]. Despite the efficient public health response to the pandemic, COVID-19 was not equally distributed among all segments of the general population. Based on our findings, individuals with a low socioeconomic status face greater challenges in dealing with the disease and have a fear of COVID-19 that is twice as high. Among the psychosocial and cultural reasons that affect the utilization of health services, there are many factors, such as knowledge, attitudes, behaviors, beliefs, traditions, and customs [29].

Another result of our research was that having chronic diseases increased fear of COVID-19. In addition, they were more affected by the possibility of getting sick and negative health beliefs. Chronic diseases have created a basis that intensifies the effect of the COVID-19 pandemic. Research has indicated that those with underlying comorbidities are more prone to experiencing severe courses of COVID-19 [30]. The studies examining the effect of patient activity on self-care in chronic disease management show that the most significant role is played by the patient. The health-seeking behavior of individuals is shaped by their health beliefs, knowledge, life skills, and motivation, all of which play a crucial role in designing effective health initiatives. The researchers define patient activity as the total of an individual’s knowledge, skill, belief/trust, and behavior [31]. Our study revealed that there was no significant association between the status of the first health institution presented to when necessary and fear of COVID-19. On the other hand, health-seeking behavior and healthy lifestyle behaviors were higher in those who presented to a family medicine unit as the first health institution. Our results showed again the importance of the family medicine unit, which is the easiest to reach in the dimension of professional health-seeking behavior.

Similar modeling studies conducted abroad mostly examined the effects of health behaviors of individuals with chronic diseases. Behavioral models are the basis of modeling studies, especially in health. In another model study that examined the relation between perceived leadership behaviors among doctors, nurses, and administrative staff and their levels of organizational commitment and the influence of organizational culture, a positive correlation was found between doctors’, nurses’, and administrative staff’s perception of interactional and transformational leadership behaviors within the healthcare group [32]. In a similar study, the structural equation model revealed a positive and meaningful impact on hope, optimism, self-efficacy, psychological resilience, service innovation behavior, and new service development to evaluate the impact of innovations to meet the desired needs of healthcare sector employees [33]. From this aspect, modeling research in the health field will provide access to more effective and valuable scientific data in taking steps toward developing health services. Our research provides access to more effective scientific data to understand the variables affecting health-seeking behaviors and their effectiveness levels for health policies in this field.

## 5. Conclusion

According to the results of our study, the participants’ health-seeking behaviors were influenced by their health perceptions and healthy lifestyle choices, and not by their level of fear towards COVID-19. From this result, health cognitions and health behaviors that have become a lifestyle were among the main factors that led to the health-seeking behavior during the COVID-19 pandemic. In the event of future public health issues caused by pandemics, this research can provide valuable insights for further research.

## 6. Limitations

Firstly, since participation in this study was voluntary, the participants had more information than expected about the research subject; for example, most of the participants were healthcare workers, which might be a limitation due to their being more knowledgeable. In the daytime, the survey study was limited as the people who were at home were mostly homemakers, the elderly, and sick people. Further, the varying effects of COVID-19 on individuals may have resulted in varying levels of anxiety at different stages of the illness.

In the present study, fit criteria of the HSBS model other than SRMR and CD did not show sufficient fit. Schermelleh-Engel et al. and Hair et al. argued that when the model was not adequately defined, it could not meet all the fit criteria [34, 35]. This might be because the scales included in the model do not meet the fit criteria or the sample size was insufficient to implement the model.

## Figures and Tables

**Figure:**
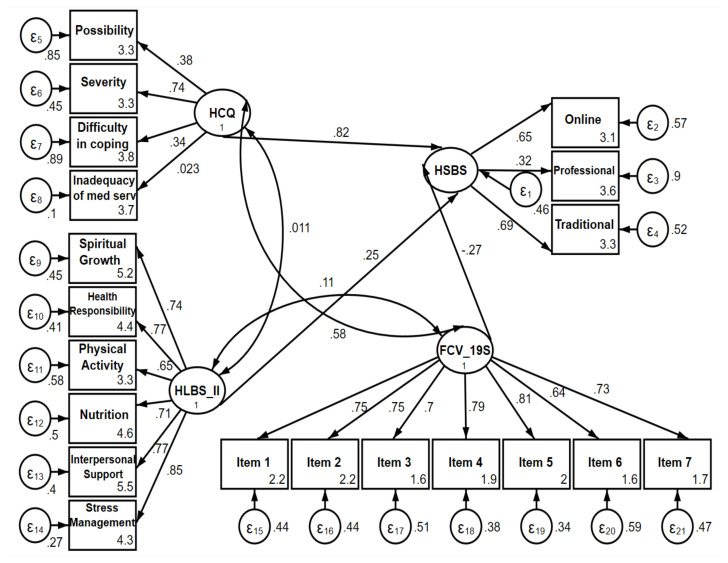
Structural equation model of health-seeking behavior. HSBS: Health Seeking Behavior Scale, HCQ: Health Cognitions Questionnaire, HLBS-II: Healthy Lifestyle Behaviors Scale-II, FCV-19S: The Fear of COVID-19 Scale. med serv: medical services.

**Table 1:** HCQ, HSBS, FCV-19S, and HLBS-II median values of score points.

Scales	n	Median (range)
HCQ	386	58 (25–87)
HSBS	386	39 (12–60)
FCV-19S	389	16 (7–35)
HLBS-II	375	137 (70–205)

HSBS: Health Seeking Behavior Scale, HCQ: Health Cognitions Questionnaire, HLBS-II: Healthy Lifestyle Behaviors Scale-II, FCV-19S: The Fear of COVID-19 Scale.

**Table 2:** Associations between scale scores and individual characteristics in univariate logistic regression analysis.^a^

Health Seeking Behavior Scale (HSBS) (score > 39)	
Living place until the age of 12^b^	Town
	District center
Educational level of mother^c^	Elementary school graduates
Educational level of father^c^	Secondary school graduates
Health Cognitions Questionnaire (HCQ) (score > 58)	
Living place until the age of 12^b^	Town
	District center
Healthy Lifestyle Behaviors Scale-II (HLBS-II) (score > 137)	
Age^d^	31–40 years
	61–70 years
Perceived socioeconomic status^e^	Above the average
Fear of COVID-19 Scale (FCV-19S) (score > 16)	
Females	
Nonhealthcare professionals	
Perceived socioeconomic status^e^	

Below low-level

a Sociodemographic and medical characteristics of the participants were included in the analysis. Scale scores were dichotomized according to the median values of the study group and considered the dependent variable in the analyses. Statistically significant results are shown in the table.

b Living in a village was the reference category.

c Illiterate was the reference category.

d <20 years was the reference category.

e Average socioeconomic status was the reference category.

OR: odds ratio, CI: confidence interval.

**Table 3:** Structural equation model findings of factors affecting health-seeking behavior.

Model HSBS	Coefficient	Standard error	p-value	95.0% CI
HCQ	2.30	0.55	<0.0001	1.23–3.37
HLBS-II	0.25	0.08	0.002	0.09–0.41
FCV-19S	−1.05	0.57	0.066	−2.16–0.07

**Table 4:** Compliance criteria and acceptable values of the HSBS model.

Compliance criteria	HSBS model	Acceptable values
Chi-squared	883.23	
Df	164	
Chi-squared/df	5.39	2 ≤ *χ*^2^ / *df* ≤ 3
p>chi-squared	<0.0001	≥0.01
RMSEA	0.11	≤0.08
CFI	0.80	≥0.95
TLI	0.76	≥0.95
SRMR	0.09	≤0.10
CD	0.99	≥0.75

HSBS: Health Seeking Behavior Scale, HCQ: Health Cognitions Questionnaire, HLBS-II: Healthy Lifestyle Behaviors Scale-II, FCV-19S: The Fear of COVID-19 Scale, RMSEA: root mean squared error of approximation, CFI: comparative fit index, TLI: Tucker–Lewis index, SRMR: standardized root mean squared residual, CD: coefficient of determination.
